# Accuracy of a faecal immunochemical test in patients under colonoscopy surveillance of colorectal adenoma and cancer

**DOI:** 10.48101/ujms.v128.8869

**Published:** 2023-06-23

**Authors:** Louise Olsson, Daniel Sjöberg

**Affiliations:** a School of Medical Sciences, Örebro University, Örebro, Sweden; b Camtö, Örebro University Hospital, Örebro, Sweden; c Centre for Clinical Research in Dalarna, Falun, Sweden

**Keywords:** Colorectal cancer, adenoma, faecal immunochemical test, surveillance, colonoscopy

## Abstract

**Background:**

Surveillance of colorectal neoplasia place great strain on colonoscopy resources, and faecal immunochemical tests (FIT) are under-investigated for this purpose. The aim of this study was to report the outcome of FIT among patients scheduled for post-polypectomy and post-resection colorectal cancer (CRC) surveillance.

**Methods:**

Patients scheduled for colonoscopy surveillance at five endoscopy units in mid-Sweden in 2016–2020 were eligible. They provided a faecal sample from 2 separate days, which were analysed by iFOBT QuikRead go® (Aidian Oy). Both the colonoscopies, and the FIT analyses were conducted by staff blinded to the other.

**Results:**

Out of 216 included patients, 157 (73%) underwent both a complete colonoscopy and had at least one FIT analysed prior to the examination. The indication for surveillance was previous adenoma in 69 (44%) and post-resection CRC in 88 (56%) patients. Two (1%) in the CRC surveillance group were diagnosed with a metachronous CRC, whereas 49 (56%) patients in the CRC surveillance, and 17 (25%) in the adenoma group had no pathology identified at colonscopy (*P* < 0.001). The proportion of patients diagnosed with adenomas requiring surveillance according to European Society of Gastrointestinal Society (ESGE) guidelines 2020 was 6 (7%) in the post-CRC resection versus 7 (10%) in the adenoma surveillance group (*P* = 0.4). Based on one FIT and at cut-off 10 µg Hb/g, sensitivity for CRC was 100%, specificity 83% (95% confidence interval [CI]: 77–89), Positive Predictive Value (PPV) 7% (−2 to 16) and Negative Predictive Value (NPV) 100%. All patients with an adenoma requiring surveillance had a FIT below this cut-off. Adding a second FIT decreased the specificity.

**Conclusion:**

Larger studies to evaluate the accuracy and consequences of using FIT for surveillance of colorectal neoplasia are needed. FIT may be more interesting for post-resection CRC surveillance than follow-up of adenoma.

## Introduction

Post-polypectomy and post-resection colorectal cancer (CRC) surveillance claim a large proportion of available colonoscopy resources, estimated to 18% of colonoscopies in the UK (1). Lately, as new guidelines state more strict criteria for adenoma surveillance (2), the number of patients included in surveillance programme due to adenomas will probably decrease (3), but on the other hand, as more individuals are included in screening programmes, the influx for surveillance will continue.

There is, therefore, a growing interest in using quantitative faecal immunochemical tests (FIT) as a triage test for colonoscopy in surveillance of adenoma patients (4). Such a surveillance regime would expose fewer patients to the discomfort of bowel cleansing and colonoscopy itself, and come at a lower cost. However, data on the consequences of using FIT, at various cut-offs for faecal haemoglobin, in patients subjected to colonoscopy surveillance specifically is still very scarce. The aim of this study was to compare the outcome of FIT in patients scheduled for colonoscopy, for post-polypectomy and post-resection CRC surveillance.

## Methods

### Participants, inclusion and sample collection

This is a prospective study on patients ≥18 years referred for colonoscopy surveillance after polypectomy of an adenoma, or radical resection of CRC at five endoscopy units (Örebro University hospital, and Falun hospital in mid-Sweden, and Södersjukhuset, Aleris Sabbatsberg hospital and Ersta hospital in Stockholm). Patients were included from June 2016 to January 2020 but recruitment periods differed between the sites and the inclusion rate varied within each site. Exclusion criteria were surveillance due to IBD, chronic radiation proctitis and hereditary syndromes Hereditary Nonpolyposis Colorectal Cancer, Adenomatous Polyposis Coli (HNPCC, APC). The dates and the quality of the previous colonoscopies were not available.

Eligible patients were contacted by phone well before their scheduled colonoscopy, and those interested in participating were mailed written study information, a consent form, sampling instructions and two FIT sampling devices (QuikRead go® FOB Sampling set, Aidian Oy, Espoo, Finland), and envelopes with pre-paid stamps. They were asked about current symptoms and medication (anticoagulants), but received no instructions on diet or the use of drugs prior to sample collection. Patients were to collect the faecal samples from 2 different days and fill in the dates of collection. They were to return the samples on the same day, or keep them in the refrigerator for at most 3 days before sending them off.

### Index test

The faecal samples were sent to Unilabs laboratory, accredited according to ISO 15189, at Eskilstuna hospital, Sweden. No clinical information accompanied the samples. The samples were analysed on the day of arrival using the QuikRead go® instrument (Aidian Oy, Finland).

This is a point-of-care quantitative immunochemical test device (5). All technicians involved received specific training for the study. A positive calibration control was done for every 50 analyses, and all were performed according to a specific standard operating protocol (SOP) from the manufacturer. During the study period, the instrument provided numerical results of haemoglobin concentration in the range 15 to >200 µg Hb/g faeces. In order to get numerical results also for the interval 10–15 µg Hb/g faeces, the manufacturer carried out supplementary analyses of the haemoglobin absorbance obtained from the instrument. Concentrations below the latter interval were reported as <10 µg Hb/g faeces. A checklist for reporting on FIT is provided in Appendix 1 (6).

### Reference standard

The overwhelming majority of colonoscopies were performed by gastroenterologists and according to routine standards. The study relied on clinical judgement insofar as any examination would be interrupted if the bowel cleansing was considered too poor. Information on all documented intraluminal findings, bowel cleansing, and completeness of the examination (intubation of caecum or ileum) was extracted from the colonoscopy reports by one or two experienced endoscopy nurses at each site. Neither the endoscopists, nor the nurses had any information on the FIT outcome. Biopsy specimens were sent for pathology assessment according to routines in the regular clinical setting and the findings were retrieved from the pathology reports.

### Categorisation of findings

The colonoscopy findings were categorized as CRC, inflammation, diverticulosis and ‘no pathology’, the first two were confirmed by pathology reports. All available data on polyps/adenomas, i.e. number of lesions, macroscopic appearance, size and pathologists´ assessment of grade of dysplasia and cellular architecture were categorized independently by both researchers (LO, DS) according to guidelines of the European Society of Gastrointestinal Endoscopy from 2020 and 2013, respectively (2, 7). The classification from 2013 involved categorisation into a low risk group (1–2 tubular adenomas < 10 mm and with low grade dysplasia) and a high risk group (adenomas with villous architecture or high grade dysplasia, ≥10 mm or ≥2 adenomas). The classification from 2020 involved adenomas requiring surveillance (at least 1 adenoma ≥10 mm or with high grade dysplasia, or ≥5 adenomas, or any serrated polyp ≥ 10 mm or with dysplasia) and adenoma in need of no surveillance (complete removal of 1–4 < 10 mm adenomas with low grade dysplasia, irrespective of villous components or any serrated polyp <10 mm without dysplasia). Data were not complete on all aspects needed for classification of all lesions and some had to be classified as ‘undetermined polyps’. Any disagreement between the researchers were resolved in consensus. If patients had several neoplastic findings, they were categorized according to the most advanced lesion.

### Statistical analysis

This is a feasibility study and no specific sample size calculation was done in advance. Outcome of first faecal sample provided by each patient was denoted as ‘one FIT’, and the highest numerical outcome of any of two analyses was denoted as ‘highest value/2 FITs’. All faecal haemoglobin concentrations are reported as µg Hb/g faeces. Cut-off was set at 10 µg Hb/g faeces, but the outcome is also reported for commonly used categories <10, 10–14.9, 15–19.9 and ≥20 µg Hb/g. These cut-offs were specified in advance. Sensitivity, specificity, positive and negative predictive values were reported with 95% confidence intervals.

The chi-square test was used to compare proportions and the Mann–Whitney and *t*-tests to compare continuous variables. All analyses were executed in SPSS 22 (IBM, Chicago, IL, USA). A two-sided *P*-value <0.05 was considered statistically significant. A checklist for reporting according to the STARD guidelines is in Appendix 2 (8). The study was approved by the Ethical Review Board in Stockholm, April 2016 (D-nr 2016/711-32).

## Results

**Figure 1:**
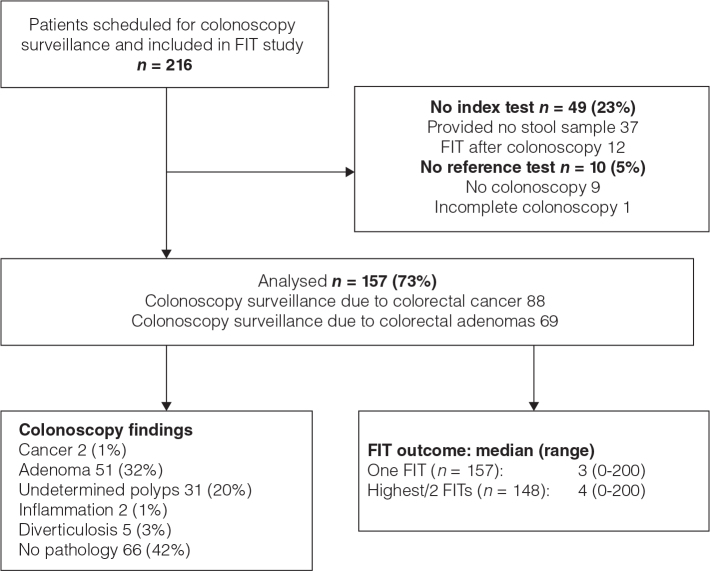
Study flow chart.

In all, 216 patients were included, but 37 patients provided no faecal sample, and 12 only after the colonoscopy (Figure 1). Ten patients provided faecal samples but nine did not undergo colonoscopy and one examination was incomplete. In total, 157 (73%) patients provided at least one stool sample and had a complete colonoscopy, and they were included in the analyses. The reason for colonoscopy was CRC surveillance for 88 (56%) and adenoma surveillance for 69 (44%) patients (Table 1).

**Table 1:** Basic characteristics of included patients (*n* = 157).

Variable	*N*	%
**Age groups (years)**		
65	50	32
66–72	53	34
73	54	34
**Sex**		
Men	90	57
Women	67	43
**Endoscopy unit**		
Sabbatsberg Aleris	13	8
Falun	33	21
Örebro	53	34
Södersjukhuset	47	30
Ersta	11	7
**Reason for surveillance**		
Cancer	88	56
Adenoma	69	44
**Reported symptoms**		
Yes	34	22
No	123	78
**Bowel cleansing**		
Complete	148	94
Some remarks	9	6
**Anticoagulants**		
Yes	17	11
No	135	86
Missing data	5	3

Median age at colonoscopy for patients undergoing CRC surveillance was 70 (range 40–85) and for adenoma surveillance 69 (range 50–88) years (*P* = 0.37). For CRC surveillance, there were 44 men and 44 women, and for adenoma surveillance, there were 46 (67%) men and 23 (33%) women (χ^2^ = 4.4; *P* = 0.04).

In all, 32 (20%) patients reported symptoms at the time of their scheduled colonoscopy (Table 1). There was no difference in the proportion reporting symptoms by indication (adenoma surveillance 16 [23%] vs. cancer surveillance 16 [18%]; *P* = 0.44), by age (above median age 20 [25%] patients vs. below median age 12 [15%; *P* = 0.12), or by sex (men 19 [21%] vs. women 13 [19%]; *P* = 0.8). The frequency of reported symptoms was as follows: diarrhoea 9 (28%), constipation 6 (19%), fresh blood 5 (16%), change of bowel habits 4 (12%), abdominal pain 3 (9%), bloating 3 (9%), and difficulties in emptying 2 (6%).

## Findings

The findings of colonoscopy are summarized in Table 2. Two patients had CRC and 66 (42%) had a clean colon. In all, 51 (32%) patients had adenomas (13 requiring surveillance and 38 requiring no surveillance) whereas for 31 (20%) patients, data extracted from the colonoscopy reports was insufficient to admit any further classification than ‘undetermined polyps’. In 24 of these 31 cases, the polyps had not been sent for microscopic evaluation, and most of these polyps were depicted as diminutive, or minimal. The size of 13 adenoma classifying for surveillance according to ESGE 2020 was median 10 (range 2–20) mm, size available for 31/38 adenoma categorized as ‘no surveillance’ was median 5 (range 1–15) and for 16/31 in the undetermined group, size was median 4 (range 1–14) mm.

**Table 2:** Distribution of colonoscopy findings by indication for surveillance (*n* = 157).

Colonoscopy findings	Cancer surveillance *n* = 88		Adenoma surveillance *n* = 69		Total *n* = 157	
	*n*	%	*N*	%	*n*	%
Cancer	2	(2)	0	(0)	2	(1)
Adenoma* and polyps^1^	34	(39)	48	(70)	82	(52)
Surveillance *	*6*	*(*7*)*	*7*	*(*10*)*	*13*	*(*8*)*
No surveillance*	*16*	*(*18*)*	*22*	*(32)*	*38*	*(*24*)*
* Undetermined* ^1^	*12*	*(*14*)*	*19*	*(*28*)*	*31*	*(*20*)*
Inflammation	1	(1)	1	(1)	2	(1)
Diverticulosis	2	(2)	3	(4)	5	(3)
No pathology	49	(56)	17	(25)	66	(42)
Total	88	(100)	69	(100)	157	(100)

* Classification according to ESGE 2020.

1 Insufficient data for further classification of these polyps.

Number in italics indicate subgroups of adenomas and polyps.

A larger proportion of patients in the post-resection CRC group had no pathology at colonoscopy compared with the adenoma surveillance group (56% vs. 25%) (χ^2^ = 16.4; *P* = 0.002) (Table 2). The adenoma surveillance group was characterized both by a larger proportion of adenoma not qualifying for surveillance (32%) and undetermined polyps (28%), but there was no difference in the proportion of adenoma qualifying for surveillance between the post-resection CRC (6/88 = 7%) and the adenoma surveillance group (7/69 = 10%), (χ^2^ = 0.6; *P* = 0.4).

Among patients with adenoma and undetermined polyps, 20/82 (24%) reported any symptom versus 12/66 (18%) among patients with no pathology detected on colonoscopy (χ^2^ = 0.8; *P* = 0.4). Neither of the two patients with CRC reported any symptom.

The findings on adenoma were compared using the ESGE 2013 and ESGE 2020 classifications and out of 24 patients categorized as high risk according to ESGE 2013, 11/24 (46%) did not fulfill the criteria for the surveillance group according to ESGE 2020 (Table 3).

**Table 3:** Classification of adenoma according to guidelines from ESGE 2020 (surveillance, no surveillance) versus ESGE 2013 (high risk, low risk) (*n* = 51).

Adenoma classification		ESGE 2020		
		Surveillance	No surveillance	Total
ESGE 2013	High risk	13 (54)	11 (46)	24 (100)
	Low risk	0	27	27 (100)
	Total	13 (25)	38 (75)	51 (100)

Values in parentheses are row percentages.

### Outcome of FIT

The number of days from collection of the first faecal sample to colonoscopy was median 13 (range 1–133). The number of days from faecal collection until analysis was median 2 (range 0–7) for both the first and second FIT. In all, 148 (94%) provided two FITs (Figure 1). Outcome of the first FIT was median 3 (range 0–200) µg Hb/g faeces, of the second FIT it was median 4 (range 0–200), and the highest value/2 FITs was median 5 (range 0–200). Among 32 patients who reported symptoms, first FIT was median 2 (range 0–35), versus median 3 (range 0–200) in 125 patients who reported no symptoms (χ^2^ = 0.1; *P* = 0.8).

The first FIT from the two patients with CRC showed 66 and 85 µg Hb/g, respectively, whereas first FIT from patients with adenomas qualifying for surveillance according to ESGE 2020 all showed <10 µg Hb/g (Table 4). Twelve patients in this adenoma group provided two faecal samples but only 1/12 had an outcome above this cut-off. For patients with no pathology at colonoscopy, first FIT was <10 µg Hb/g in 58 (88%).

**Table 4:** Outcome of one FIT and max value/ two FITs (µg Hb/g) by colonoscopy findings.

Colonoscopy findings	9.9	10–14.9	15–19.9	≥20	Total
**One FIT**					
Cancer	0	0	0	2 (100)	2
Adenoma* and polyps^1^	65 (79)	5 (6)	3 (4)	9 (11)	82
* Surveillance* *	*13 (100)*	*0*	*0*	*0*	*13*
* No surveillance* *	*31 (82)*	*3 (*8*)*	*2 (*5*)*	*2 (*5*)*	*38*
* Undetermined* ^1^	*21 (68)*	*2 (*6*)*	*1 (*3*)*	*7 (*23*)*	*31*
Inflammation	1 (50)	0	1 (50)	0	2
Diverticulosis	4 (80)	0	1 (20)	0	5
No pathology	58 (88)	1 (2)	1 (2)	6 (9)	66
Total	128 (82)	6 (4)	6 (4)	17 (11)	157 (100)
**Max value/two FITs**					
Cancer	0	0	0	2 (100)	2
Adenoma* and polyps^1^	58 (73)	6 (8)	4 (5.0)	12 (15)	80
* Surveillance* *	*11 (92)*	*1 (*8*)*	*0*	*0*	*12*
* No surveillance* *	*26 (70)*	*3 (*8*)*	*3 (*8*)*	*5 (*14*)*	*37*
* Undetermined* ^1^	*21 (68)*	*2 (*6*)*	*1 (*3*)*	*7 (*23*)*	*31*
Inflammation	1 (50)	0	0	1 (50)	2
Diverticulosis	4 (80)	0	1 (20)	0	5
No pathology	44 (75)	5 (8)	3 (5)	7 (12)	59
Total	107 (72)	11 (7)	8 (5)	22 (15)	148 (100)

FIT: faecal immunochemical tests.

* Adenoma classification according to ESGE 2020.

1 Insufficient data for further classification of these polyps.

Values in parenthesis are row percentages.

Based on one FIT and cut-off 10 µg Hb/g for positivity, 29/157 (18%) colonoscopies would have been carried out, both CRC identified, and all 13 adenoma for surveillance missed. Based on the highest value/2 FITs and cut-off at 10 µg Hb/g for positivity, 41 (28%) colonoscopies would have been carried out, both CRC identified and 11/12 (92%) adenoma for surveillance would have been missed. A summary of the calculated accuracy is in Table 5. Higher cut-off values increased specificity, but adding a second FIT decreased specificity.

**Table 5:** Accuracy and predictive values for colorectal cancer of one FIT and max value/2 FITs at cut-off 10, 15 and 20 µg Hb/g faeces.

One FIT	Cut-off	Sensitivity	Specificity (95% CI)	PPV (95% CI)	NPV
	≥10	100	83 (77–89)	7 (−2 to 16)	100
	≥15	100	86 (81–92)	9 (−3 to 20)	100
	≥20	100	90 (86–95)	12 (−4 to 27)	100
Highest/2 FITs	≥10	100	73 (66–80)	5 (−2 to 11)	100
	≥15	100	80 (74–87)	7 (−2 to 16)	100
	≥20	100	86 (81–92)	9 (−2 to 21)	100

FIT: faecal immunochemical tests; CI: confidence interval; PPV: positive predictive value; NPV: negative predictive value.

### Budget impact

The estimated cost for one colonoscopy in Sweden, 2022 is approximately 790 € (9). The cost for all 157 scheduled surveillance colonoscopies equals (157 × 660) 124,030 €. For one FIT and cut-off at 10 µg/g, 128/157 (82%) were negative, theoretically lowering the costs for colonoscopy by (128 × 790) 101,120 €. The costs for the colonoscopies would have been 22,910 € to detect 2/2 CRC and 0/13 adenomas qualifying for surveillance. An estimated total cost for one FIT of 10–20 € would add another 1,570–3,140 € to the total costs. This would entail a reduction of costs close to 80% for surveillance in this particular group.

## Discussion

This study on post-polypectomy and post-resection CRC surveillance, the first FIT of both of the two patients diagnosed with CRC had high concentrations of faecal haemoglobin well above cut-off at 10 µg/g faeces, whereas more than 80% of patients had a negative (<10 µg/g faeces) test. This included 13 patients diagnosed with adenomas qualifying for surveillance.

There is only a limited number of studies on FIT for surveillance of colorectal neoplasia available. For adenoma surveillance specifically, Cross et al. recruited 6,000 patients after a positive gFOBT and follow-up colonoscopy, as part of a screening programme in southern England in 2012–2013 (10). After polypectomy, patients with an intermediate risk of CRC (3–4 small adenomas or one adenoma ≥10 mm) had annual FIT (OC-Sensor DIANA) and a colonoscopy after 3 years. Overall programme sensitivity of FIT at cut-off 10 μg/g was 72% for CRC and 57% for advanced adenoma (AA); 3-year positivity at this cut-off was 29%. In other words, replacing a colonoscopy surveillance after 3 years with annual FIT could reduce colonoscopies by 71%, but would miss 30–40% of CRCs and a large proportion of AA. But, importantly, sensitivity for CRC of first FIT in this study was 52% (95% confidence interval [CI]: 32–71), and 33% (95% CI: 29–38) for AA.

A prospective, double-blind study from Israel published in 2010 included 1,071 consecutive, asymptomatic patients scheduled for colonoscopy surveillance after resection of CRC, or adenoma polypectomy, or at increased risk of CRC due to family history (11). Participants provided three stool samples for OC-MICRO I-FOBT analysis. First test was positive in 8%, cumulative positivity of the first two tests rose to 12%, and was 15% of all three tests. At the lowest threshold of 50 ng Hb/mL of buffer, and using only the first I-FOBT, sensitivity for CRC was 100% and 65% for all significant neoplasms (CRC or advanced adenomatous polyps). Finally, a study from the Netherlands conducted in 2006–2009 including 1,041 participants scheduled for colonoscopy surveillance due to personal history of adenoma/CRC, or family history of CRC, reported an overall positivity rate of 11% at cut-off 50 ng/mL using OC-Sensor (12). Sensitivity for CRC was 80% (95% CI: 28–99) and 28% (95% CI: 19–38) for advanced adenoma. This study is the first to report a sensitivity of 100 and 0% for CRC and AA, respectively, of one FIT.

The intensity of colonoscopy surveillance of colorectal adenoma has been reduced lately, and one specific example is the previously recommended colonoscopy surveillance at intervals of 3 years for individuals with an estimated intermediate risk of CRC. For this group, Atkin et al. found that, if baseline colonoscopy was of high quality, and if there were no proximal polyps, high-grade or large adenoma ≥20 mm, the risk of CRC was even lower than that of the general population (13). Since 2020, ESGE has adopted more strict criteria for colonoscopy surveillance after 3 years and it is now recommended when at least one adenoma ≥10 mm, or with high‐grade dysplasia, or ≥5 adenomas, or any serrated polyp ≥10 mm or with dysplasia is detected, i.e. villous architecture and merely three adenoma are no longer indications for surveillance (2). A study from Austria contrasted these new guidelines with the 2013 recommendations (7) and, as part of a quality assurance programme, found the proportion of individuals assigned to 3-year colonoscopy fell from 10.4 to 4.9%, a relative reduction of 47% (3). This is very close to our findings in this study (11/24; 46%). The Austrian researchers also reported maintained, or even improved, risk stratification because the point estimate of CRC mortality was higher in the surveillance group according to 2020 guidelines compared with the high-risk group adenoma of guidelines from 2013 (Hazard ratio (HR): 2.6; 95% CI: 1.6–4.0 vs. HR: 1.7; 95% CI: 1.1–2.6). In addition, there was no difference in all-cause mortality between the no surveillance group of 2020 guidelines as compared with low-risk group of 2013 (HR: 1.06, 95% CI: 1.01–1.11 vs. HR: 1.05, 95% CI: 0.99–1.10). This clearly indicates that there has been an overuse of colonoscopy surveillance.

Concerning surveillance after resection of CRC, a systematic review and meta-analysis reported a cumulative incidence of metachronous CRC over 16 years of 2.2% (95% CI: 1.8–2.9), the overwhelming majority being detected within the first 36 months (14). In addition, the cumulative incidence of anastomotic CRC was 2.7% (95% CI: 1.9–3.9). The literature search included studies from inception and up to 2018, and only a smaller part provided data from the era of colonoscopies of high quality. However, even in a recent study of Dutch patients who had undergone preoperative colonoscopies in 2013–2016, five metachronous CRC, and five anastomotic recurrences (10/572 ~1.7% lesions) were detected at colonoscopy after mean 13 months (15). In all, there is limited evidence underpinning current guidelines on colonoscopy surveillance after CRC, but colonoscopy is in general recommended 1 year after resection, and then after 3 and 5 years (16).

Adherence to guidelines on colonoscopy surveillance intervals is a well-known problem and estimated to merely 49% (17, 18) and shorter intervals are often recommended by physicians (19). Interestingly, the study outlined here on surveillance of adenoma with an estimated intermediate risk (13) was made possible only due to the fact that 5,019/11,944 (42%) eligible patients did *not* attend surveillance, compared with 6925 (58%) individuals who complied with the recommendations. Common reasons among patients are fear of pain and discomfort, and ‘concerns about bowel preparation’ (20). In hypothetical scenarios presented to the English public, FIT was preferred over colonoscopy for both adenoma surveillance, and work-up of symptoms (21, 22).

Incremental costs per additional advanced adenoma detected by colonoscopy versus by FIT at cut-off 10 μg/g was estimated to £8,863 (95% CI: 7,018–10,939) and per additional CRC to £243,094 (95% CI: −1,242,531 to 1,990,865) in the largest study on FIT for surveillance so far by Cross et al. (10). A simulation study based on the Dutch screening programme among asymptomatic individuals of 55 to 75 years found that adding colonoscopy surveillance to FIT screening was not cost-effective based on the Dutch ICER threshold, and increased the colonoscopy demand substantially (23). Both examples illustrate the resource-consuming aspect of primarily colonoscopy-based surveillance.

Limitations of this study are the low number of participants of a convenience series. Merits are the blinded collection of data, a meticulous classification of adenomas/polyps, and the comparison of FIT surveillance of adenoma and CRC in a setting representing routine care. Overall, our findings are consistent with previous studies, i.e. there is a significant difference in the ability of FIT to detect CRC and adenoma (24). However, a few circumstances underline FIT surveillance as an important topic for further studies. Firstly, there is little hard evidence on the effect of colonoscopy surveillance on CRC incidence and CRC mortality, and the reduction of CRC risk compared with the general population was quite recently shown to be limited to high-risk groups only (25). It has also been put forward that surveillance may be limited to adenoma sized ≥20 mm or high-grade dysplasia, in particular for healthcare systems with limited capacity, as this would reduce the number of colonoscopies substantially with little effect on CRC mortality (26). The findings were also reproduced by the Austrian researchers (3). This is interesting, as FIT level is associated with adenoma size (27). Secondly, adherence to surveillance is crucial, and FIT may make up a first option for patients who hesitate to undergo colonoscopy or bowel cleansing for psychological reasons, for patients with severe comorbidity, or for those living far away from nearest endoscopy unit (28). Thirdly, health economic aspects favour FIT surveillance, and fourthly, free up the colonoscopy resource for symptomatic patients or screening. Another possibility is to integrate FIT into existing colonoscopy surveillance programmes to prolong or personalize the colonoscopy intervals (29).

In all, we conclude further studies on FIT surveillance of colorectal neoplasia are warranted.

## Appendix 1. Checklist for reporting on faecal immunochemical tests.

**Table:**

Specimen collection and handling		
QuikRead go® Aidian Oy, Råsta Strand väg 13 C 169 79 Solna	Name of specimen collection device and supplier (address).	Essential
Quik Read FOB probe	Description of specimen collection device (vial with probe/stick, card, other).	Essential
Single faecal samples	Description of specimens used if an *in vivo* study (single or pooled faeces, artificial matrix with added blood, etc.).	Essential for laboratory evaluations
Probe	Details of faecal collection method (sampling technique and number of samples).	Essential
Patients	Who collected the specimens from the samples (patient, technician, etc.).	Essential
Two faecal specimens from two separate days	Number of faecal specimens used in the study (single, pooled, individual patient faeces).	Essential for laboratory evaluations
10 mg	Mean mass of faeces collected.*	Essential
Buffer into which specimen is taken by probe	Volume of buffer into which specimen is taken by probe, applicator stick or card.*	Essential
Kept at most 3 days in fridge temperature at home before sending to the laboratory by ordinary post (1 day)	Time and storage conditions of faecal specimen from ‘passing’ to sampling, including time and temperature (median and range).	Essential for laboratory evaluations
Specimen were analysed on the day of arrival to the laboratory	Time and storage of collection devices from specimen collection to analysis, including time and temperature (median and range). A concise description of process from collection to analysis is recommended.	Essential

**Table:**

Analysis		
QuikRead go® Aidian Oy, Råsta Strand väg 13 C 169 79 Solna Tel +468-623 04 00 One instrument was used during the study	Name of analyser, model, supplier (address), number of systems if more than one used.	Essential
One	Number of times each sample was analysed.	Essential
75–1000 ng Hb/mL buffer, 15–200 µg Hb/g faeces No reassays or dilutions	Analytical working range* and whether samples outside this range were diluted (factor) and reassayed.	Essential for laboratory evaluations
QuikRead go iFOBT. The reference material has been verified with a method comparable to the international Council of Standardisation in Haematology (ISCH) reference method.	Source of calibrator(s) (supplier with address), number of calibrator(s), how concentrations were assigned* and details of calibration process including frequency.	Essential for laboratory evaluations
~ 340 samples analysed. Mean (SD) was 11,2 (30,4) µg Hb/g faeces.	Analytical imprecision*, ideally with number of samples analysed, concentrations, and mean, SD and CV.	Essential for all studies

**Table:**

Quality management		
Quik Read FOB positive control Cat.No 06027 Hb in buffert >1000 ng/mL or > 200 μg Hb/g faeces. One control performed per kit (50 tests)	Source (address) or description of internal quality control materials, number of controls, assigned target concentrations and ranges, how target concentrations were assigned, rules used for acceptance and rejection of analytical runs.	Desirable for laboratory evaluations
No	Participation in external quality assessment schemes: (name and address of scheme), frequency of challenges, performance attained.	Desirable for laboratory evaluations
Unilabs Inc, Dep of Clinical Chemistry, Mälarsjukhuset S-631 88 Eskilstuna Sweden	Accreditation held by the analytical facility (address).	Desirable for laboratory evaluations
5 biomedical scientists	The number, training and expertise of the persons performing the analyses and recording the results.	Essential

**Table:**

Result handling		
Electronic and manual recording, single reading	Mode of collection of data – manual recording or via automatic download to IT system, single or double reading.	Desirable
μg Hb/g faeces	Units used, with conversion to μg Hb/g faeces if ng Hb/mL used.	Essential
Assigned by instrument 15 μg Hb/g faeces and then complementary analyses by the manufacturer	Cut-off concentration(s) if used and explanation of how assigned locally or by manufacturer.*	Essential
Yes	Were the analysts blinded (masked) to the results of the reference investigation and other clinical information.	Essential

## Appendix 2. STARD guidelines for reporting diagnostic accuracy studies.

**Table:**

Section & Topic	No	Item	Reported on page #
**TITLE OR ABSTRACT**			
	**1**	Identification as a study of diagnostic accuracy using at least one measure of accuracy (such as sensitivity, specificity, predictive values, or AUC)	2
**ABSTRACT**			
	**2**	Structured summary of study design, methods, results, and conclusions (for specific guidance, see STARD for Abstracts)	2
**INTRODUCTION**			
	**3**	Scientific and clinical background, including the intended use and clinical role of the index test	3
	**4**	Study objectives and hypotheses	3
**METHODS**			
*Study design*	**5**	Whether data collection was planned before the index test and reference standard were performed (prospective study) or after (retrospective study)	4
*Participants*	**6**	Eligibility criteria	4
	**7**	On what basis potentially eligible participants were identified (such as symptoms, results from previous tests, inclusion in registry)	4
	**8**	Where and when potentially eligible participants were identified (setting, location and dates)	4
	**9**	Whether participants formed a consecutive, random or convenience series	4, 14
*Test methods*	**10a**	Index test, in sufficient detail to allow replication	4
	**10b**	Reference standard, in sufficient detail to allow replication	5
	**11**	Rationale for choosing the reference standard (if alternatives exist)	-
	**12a**	Definition of and rationale for test positivity cut-offs or result categories of the index test, distinguishing pre-specified from exploratory	6
	**12b**	Definition of and rationale for test positivity cut-offs or result categories of the reference standard, distinguishing pre-specified from exploratory	6
	**13a**	Whether clinical information and reference standard results were available to the performers/readers of the index test	4
	**13b**	Whether clinical information and index test results were available to the assessors of the reference standard	5
*Analysis*	**14**	Methods for estimating or comparing measures of diagnostic accuracy	7
	**15**	How indeterminate index test or reference standard results were handled	8, Figure 1
	**16**	How missing data on the index test and reference standard were handled	8, Figure 1
	**17**	Any analyses of variability in diagnostic accuracy, distinguishing pre-specified from exploratory	7
	**18**	Intended sample size and how it was determined	6
**RESULTS**			
*Participants*	**19**	Flow of participants, using a diagram	Fig 1
	**20**	Baseline demographic and clinical characteristics of participants	Table 1
	**21a**	Distribution of severity of disease in those with the target condition	Table 1
	**21b**	Distribution of alternative diagnoses in those without the target condition	Table 1
	**22**	Time interval and any clinical interventions between index test and reference standard	9
*Test results*	**23**	Cross tabulation of the index test results (or their distribution) by the results of the reference standard	Table 4
	**24**	Estimates of diagnostic accuracy and their precision (such as 95% confidence intervals)	Table 5
	**25**	Any adverse events from performing the index test or the reference standard	Not reported
**DISCUSSION**			
	**26**	Study limitations, including sources of potential bias, statistical uncertainty, and generalisability	12–16
	**27**	Implications for practice, including the intended use and clinical role of the index test	12–16
**OTHER INFORMATION**			
	**28**	Registration number and name of registry	7
	**29**	Where the full study protocol can be accessed	-
	**30**	Sources of funding and other support; role of funders	16
